# Determinants of poorly controlled asthma among asthmatic patients in Jimma University Medical Center, Southwest Ethiopia: a case control study

**DOI:** 10.1186/s13104-019-4571-y

**Published:** 2019-08-20

**Authors:** Ameha Zewudie, Tadesse Nigussie, Yitagesu Mamo, Kabaye Kumela

**Affiliations:** 1 Department of Pharmacy, College of Health Science, MizanTepi University, Mizan-Aman, Ethiopia; 2 Department of Public Health, College of Health Sciences, MizanTepi University, Mizan-Aman, Ethiopia; 3 Department of Pharmacy, Institute of Health Science, Jimma University, Jimma, Ethiopia

**Keywords:** Asthma, Poorly controlled asthma, Ethiopia

## Abstract

**Objective:**

The aim of this study was to assess determinants of poorly controlled asthma among asthmatic patients on follow up at Jimma University Medical Center, Southwest Ethiopia. A facility based case control study involving chart review and patient interview was conducted from April 01/2017 to May 30/2017. Consecutive sampling method was used to select 121 cases and 121 controls. Descriptive statistics were used to present socio demographic data and drug prescription pattern while logistic regression was used to identify predictors of poorly controlled asthma.

**Results:**

From a total of 242 studied asthmatic patients, 52.9% of controls and 44.6% of cases were males. Poor knowledge about asthma [Adjusted odd ratio(AOR) = 7.30; 95% confidence interval (CI) 1.72–30; P = 0.007], negative attitude about asthma [AOR = 5.10; 95% CI 1.40–18.7; P = 0.014], moderate asthma [AOR = 13.47; 95% CI 2.69–47.23; P = 0.002] and non-adherence to inhaled corticosteroid (ICS) [AOR = 8.52%; 95% CI 2.41–13.45; P = 0.001] were determinants of poorly controlled asthma.

**Electronic supplementary material:**

The online version of this article (10.1186/s13104-019-4571-y) contains supplementary material, which is available to authorized users.

## Introduction

Worldwide, approximately 180,000 deaths annually are attributable to asthma. There has been a sharp increase in the global prevalence, morbidity, mortality and economic burden associated with asthma over the last 40 years. Asthma can place considerable limitations on the physical, emotional, social and professional lives of sufferers. These may be greater when symptoms are not adequately controlled [1].

Economic burden of asthma is also considerable. In the United States, the total direct medical and indirect economic costs of asthma were approximately $12 billion in 1994 [2]. In Europe, the total cost of asthma currently covers at approximately $21.65 billion per year [1]. In developing countries, annual asthma cost was estimated at $20 billion [3].

The control of asthma symptom is a realistic goal. Studies have shown that this can be achieved in most asthmatic patients which lead to a higher quality of life. In spite of this, the control of asthma is generally poor. The Asthma Insights and reality in Europe study reported persistence day time symptoms of up to 46% among asthmatic patients under treatment [4]. Ethiopia is not an exception. Studies done in Jimma University Medical Center (JUMC) reported that greater than half of asthmatic patients had poorly controlled asthma [5, 6].

To solve this problem, World Health Organization (WHO) developed guideline for chronic diseases management including asthma and the development of questionnaire tools to measure asthma control. One such tool is the asthma control test (ACT) which is based on 5 items survey that assess interference with activity, shortness of breath, nocturnal symptom, rescue medication use and self rating of asthma symptoms [7].

Regardless of these interventions, asthma is poorly controlled all over the world, particularly in developing countries. Hence, this study aimed at identifying predictors of poorly controlled asthma among asthmatic patients on follow up at JUMC.

## Main text

### Methods

The study was conducted at JUMC, which is found in Jimma town, Southwest Ethiopia. Hospital based unmatched case–control study was conducted from April 01/2017 to May 30, 2017 G.C. The source population was all adult asthmatic patients who were on follow up at JUMC. The study population consists of asthmatic patients attending chest clinic during the study period and fulfill inclusion criteria. Patients who had at least 3 months consecutive follow up and age 18 years or older were included. Patients with lung cancer, congestive heart failure, chronic obstructive pulmonary disease, pulmonary hypertension and interstitial lung disease were excluded due to similarities in symptoms with asthma which make the assessment of asthma severity more difficult. Mentally unstable or critically ill patients were also excluded.

Controls were patients with well controlled asthma (average short acting B-agonist (SABA) consumption ≤ 2 days per week for three consecutive months), no night awaking for three consecutive months and no emergency visits or oral courses of steroids during the past 6 months, whereas patients with poorly controlled asthma (average SABA consumption > 2 days per week for three consecutive months) or if ≥ 1-night awakenins per week for three consecutive months, or any emergency visits or oral courses of steroids during the past 6 months were considered as cases [4, 6, 8].

Asthma severity was assessed by Global Initiative for Asthma (GINA) asthma symptom control assessment tool [8], which has been correlated to other standardized asthma severity score [1]. Adherences to controllers were assessed by using Morisky 8 item medication adherence questionnaires. Accordingly, high adherence is considered if the patient score 0, medium adherence if score is 1–2 and low adherence if score is > 2. Medium adherence and low adherence were considered to be non-adherent [9, 10]. Patients knowledge about Asthma was assessed based on 20 asthma knowledge questions. Participants who achieved knowledge score ≤ 8 were considered as having poor knowledge, 9–12 as average knowledge and ≥ 13 as good knowledge. Average knowledge and good knowledge was considered as good knowledge [11, 12]. Attitude towards Asthma was evaluated with five-point Likert scale ranging from strongly disagrees to strongly agree. Total score of attitude domain ranged from 5 to 25. Patient’s attitudes were categorized into two groups, positive attitude if the score was ≥ 15 and negative attitudes if the score was < 15 [12].

Epi info version 7 was used to calculate sample size by two population proportion with the assumption of 95% CI 80% power, and 1:1 case to control ratio. OR and proportion of different determinant variables of asthma control among control was taken from recent study done at JUMC [5, 6]. After calculation, smoking gave the largest sample size which is 242. Consecutive sampling method was applied until the required sample size of both of the groups achieved.

Semi-structured questionnaires and check lists were used to collect data from the patients and their medical record respectively. The data entry was made by using Epidata manager 4.0.2.101 and statistical analysis was done by statistical Package for Social Science (SPSS) version 21. Descriptive statistics such as mean, median and standard deviations (SD) was used to summarize the results. All independent variables which have P value of less than 0.25 on binary logistic regression were selected as candidate for multivariable regression analysis. Then, multivariable logistic regression using back ward selection method was done to identify predictors of poorly controlled asthma at P value < 0.05.

### Results

#### Socio demographic characteristics of the study participants

One hundred twenty-one controls and 121 cases were included in the study. Of these, 112 (92.6%) of the controls and 35 (28.9%) of the cases had good knowledge about asthma. Eighty-nine (73.6%) of the controls and 53 (43.8%) of the cases had positive attitude towards asthma. Eighty-six (84.4%) of controls and 13 (19.1) of cases were non adherent to ICS (Table 1).

**Table 1:** Patient characteristics of the Asthmatic patients on follow up at Jimma University Medical Center, Southwest Ethiopia, 2017

Variables	Control		Case	
	Frequency	%	Frequency	%
Sex				
Male	64	52.9	54	44.6
Female	57	47.1	67	55.4
Age of the respondent (years)				
18–34	40	33.1	16	13.2
35–54	52	43.0	34	28.1
> 55	29	24.0	71	58.7
Residence				
Urban	72	59.5	42	34.7
Rural	49	40.5	79	65.3
Marital status				
Single	12	9.9	8	6.6
Married	97	80.2	83	68.6
Divorced	4	3.3	9	7.4
Widowed	8	6.6	21	17.4
Educational status				
Higher	22	18.2	11	9.1
Secondary	19	15.7	10	8.3
Primary	37	30.6	26	21.5
Illiterate	43	35.5	74	61.2
Occupational status				
Government employee	47	39.2	20	16.5
Merchant	12	10	8	6.6
Housewife	8	6.7	14	11.6
Farmer	38	31.7	47	38.8
Retired	6	5.0	25	20.7
Others	10	8.2	7	5.8
Monthly income				
≤ 1200	43	35.5	88	72.7
1201–2499	25	20.7	15	12.4
≥ 2500	53	43.8	18	14.9
Smoking status				
Never	108	89.3	68	56.2
Previously	9	7.4	37	30.6
Current	4	3.3	18	13.2
Residence				
Urban				
Rural				
Chat chewing				
Yes	24	19.8	28	23.1
No	97	80.2	93	76.9
Drinking alcohol				
Yes	116	95.9	105	86.8
No	5	4.1	16	13.2
Visiting the doctor				
Scheduled	109	90.1	95	84.3
Unscheduled	12	9.9	26	15.7
Knowledge about asthma				
Good	112	92.6	35	28.9
Poor	9	7.4	86	71.5
Attitude about asthma				
Positive attitude	89	73.6	53	43.8
Negative attitude	32	26.4	68	56.2
ICS				
Yes	90	74.4	68	56.2
No	31	25.6	55	43.8
Level of adherence to ICS				
Adherent	76	84.4	13	19.1
Non adherent	14	15.6	55	80.9

Others: unemployed, private workers but not merchant, student

#### Diseases related factors

According to the classification by GINA, 58 (47.9%) of the cases and 39 (32.2%) of the controls had moderate persistent asthma while 58 (47.9%) of the cases and 9 (7.4%) of the controls had severe persistent asthma (Table 2).

**Table 2:** Diseases related factors among Asthmatic patients on follow up at Jimma University Medical Center, Southwest Ethiopia, 2017

Variables	Control		Case	
	Frequency	%	Frequency	%
Severity of the disease				
Mild	70	57.9	5	4.1
Moderate	42	37.9	58	47.9
Severe	9	7.4	58	47.9
Follow up in years				
< 5 years	64	52.9	33	27.3
5–10 years	46	38.0	52	43.0
> 10 years	11	9.1	36	29.8
Comorbid conditions				
Yes	8	6.6	39	32.2
No	113	93.4	82	67.8
Types of comorbid conditions				
Depression	1	12.5	15	38.5
Allergic rhinitis	3	37.5	7	17.9
HTN (hypertention)	2	25.0	16	41.0
DM (diabetic mellitus)	1	12.5	5	12.8
Others^a^	2	25.0	4	10.3
Precipitating diseases				
Yes	24	19.8	44	36.4
No	97	80.2	77	63.6
Types of precipitating diseases				
GERD (gastro-esophagial reflex diseases or PUD (peptic ulcer diseases)	14	58.3	41	93.2
CAP (community acquired pneumonia)				
Or URI (upper respiratory infection)	25	33.3	11	25.3
UTI (urinary tract infection)	4	16.7	2	4.5
Any pain	1	4.2	4	9.1
Asthma exacerbation				
Yes	9	7.4	90	80.24
No	112	92.6	24	19.8
Hospitalization				
Yes	1	0.8	15	12.4
No	120	99.2	106	87.6
Cause of hospitalization				
Asthma	0	0.0	12	80.0
Others^b^	1	100	3	20.0

^a^Thyroid disorder, hepatitis, retro viral infection

^b^Pneumonia, peptic ulcer diseases

#### Predictors of poorly controlled asthma

Poor knowledge towards asthma, negative attitude towards asthma, moderate and severe asthma and non-adherence to ICS were independent predictors of poorly controlled asthma (Table 3).

**Table 3:** Predictors of poorly controlled asthma among asthmatic patients in Jimma University Medical Center, Southwest Ethiopia, 2017

Variables	Control N = 121	Case N = 121	AOR (95% CI)	*P* value
	N (%)	N (%)		
Age				
18–34	40 (33.1)	16 (13.2)	1.00	
35–54	52 (43.0)	34 (28.1)	0.63 (0.14–2.84)	0.554
> 55	29 (24.0	71 (58.7)	1.03 (0.20–.5.26)	0.974
Residence				
Urban	72 (59.5)	42 (34.7)	1.00	
Rural	49 (40.5)	79 (65.3)	1.25 (0.94–15.23)	0.1901
Smoking status				
Never	108 (89.3)	68 (56.2)	1.00	
Past	9 (7.4)	37 (30.6)	0.99 (0.15–6.4)	0.475
Current	4 (3.3)	16 (13.2)	2.54 (0.94–13.2)	0.511
Asthma knowledge				
Good	112 (92.6)	35 (28.9)	1.00	
Poor	9 (7.4)	86 (71.1)	7.30 (1.72–30.85)	0.007
Asthma attitude				
Positive	89 (73.6)	53 (43.8)	1.00	
Negative	32 (26.4)	68 (56.2)	5.10 (1.40–18.7)	0.014
Severity of asthma				
Mild	70 (57.9)	5 (4.1)	1.00	
Moderate	42 (37.9)	58 (47.9)	13.47 (2.69–47.23)	0.002
Severe	9 (7.4)	58 (47.9)	19.98 (7.03–78.89)	< 0.001
Comorbidity				
No	113 (93.4)	82 (67.8)	1.00	
Yes	8 (6.6)	39 (32.2)	3.27 (0.71–14.9)	0.126
ICS				
Yes	90 (74.4)	68 (56.2)	1.00	
No	31 (25.6)	55 (43.8)	2.5 (0.95–2.3)	0.254
Level of adherence to ICS				
Adherent	76 (84.4)	13 (19.1)	1.00	
None adherent	14 (15.6)	55 (80.9)	8.52 (2.41–13.45)	0.001

### Discussion

The present study showed that patients who had moderate and severe persistent asthma were found to be 13 and 20 times more likely to have uncontrolled asthma respectively. This finding is comparable with the study done in JUMC which reported that poorly controlled asthma were about 6 times and 32 times more likely among patients with moderate and severe asthma respectively [6]. Similarly, study conducted in Brazil revealed that patient with persistent severe asthma were 5 times more likely to have uncontrolled asthma (OR = 5.33, P < 0.0001) [13]. Another study done in Saud Arabia also demonstrated that severity of asthma significantly affects asthma control [14].

The finding of this study demonstrated patients who had poor knowledge about asthma were about 7 times increased odds of poorly controlled asthma. This finding is in line with other studies done by Mahendra et al., and study conducted in Sri Lanka [12, 15]. However, study done by Van Dellen et al. reported knowledge of patients about their disease had no association with Asthma control (OR = 1.1; 95% CI 0.71–1.83) [16]. This difference might be due to socio-demographic difference between the two populations. In addition to this, the current study was done only in a single institution while study conducted by Van Dallen et al. was multicenter.

The present study illustrated, patient negative attitude was a risk factor for poorly controlled asthma. Asthma patients with negative attitude are about 5 times more likely to had poorly controlled asthma than those patients with positive attitude. This study is in line with the study conducted by Mahendra et al. and study conducted in University of Ruhuna, Sri Lanka [12, 15].

The finding of this study revealed that non-adherence to ICS was significantly associated with poorly controlled Asthma. Similarly, other studies reported that non adherence to ICS was an independent predictor of asthma-related hospitalization [9, 17]. In addition to this, study done by Clatworthy et al. reported that low adherence to ICS was associated with poor asthma control (OR = 1.35; 95% CI 1.18–1.55) [18]. But, our finding is inconsistent with study conducted in Netherland which reported non adherence to ICS was not associated with uncontrolled asthma (OR = 0.6; 95% CI 0.38–1.07) [16]. This difference might be due to the fact that in the present study, poor knowledge and negative attitude about asthma was observed. This might affect drug taking behavior of the patients.

### Conclusion

Poor knowledge about asthma, negative attitude about asthma, moderate and severe asthma and non-adherence to inhaled corticosteroid were independent predictors of poorly controlled asthma (Additional file 1).

## Limitation of the study

Patient self-reporting of asthma control to either the physician or to the interviewer could intro duce misclassification bias by either categorizing cases as controls and controls as cases.

## Additional file

**Additional file 1.** Individual anti-asthmatic and concurrent medication related factors among asthmatic patients who have follow up at Jimma University Medical Center. This data shows anti-asthmatic and concurrent medication pattern among asthmatic patients in Jimma University Medical Center.

## Data Availability

All data generated during and/or analyzed during the study are available from the corresponding author on reasonable request.

## Abbreviations

ACT: asthma control test
CI: confidence interval
CAP: community acquired kneumonia
GERD: gastro-esophagial reflex diseases
GINA: global initiative for asthma management and prevention
ICS: inhaled corticosteroid
JUMC: Jimma University Medical Center
LABA: long acting beta_2_ agonist
AOR: adjusted odd ratio
OR: odd ratio
PUD: peptic ulcer disease
SABA: short acting beta_2_ agonist
SD: standard deviation
SPSS: Statistical Package for Social Science
URI: upper respiratory infection
UTI: urinary tract infection
WHO: World Health Organization
