# Supplementary material for: Determinants of poorly controlled asthma among asthmatic patients in Jimma University Medical Center, Southwest Ethiopia: a case control study
Source: BMC Res Notes. 2019 Aug 20;12:525. doi: 10.1186/s13104-019-4571-y (PMC6700762; doi:10.1186/s13104-019-4571-y)
Supplement: Supplementary file 1 — Additional file 1. Individual anti-asthmatic and concurrent medication related factors among asthmatic patients who have follow up at Jimma University Medical Center. This data shows anti-asthmatic and concurrent medication pattern among asthmatic patients in Jimma University Medical Center. [file 13104_2019_4571_MOESM1_ESM.docx]

**Individual anti-asthmatic and concurrent medication related factors**

ICS (Beclometasone) were prescribed in 64.9% of asthmatic patients, 68(56.2%) of the cases and 89(73.6%) of the controls. Among the patients prescribed with ICS, 13(19.1%) of the cases and 74(82.2%) of the controls were adherent to beclometasone.

**Table S1**: Individual anti-asthmatic and concurrent drugs among study participants in Jimma University Medical Center, South West Ethiopia, 2017

| Variables | | Control | | Case | | Total | |
| --- | --- | --- | --- | --- | --- | --- | --- |
|  |  | Frequency | % | Frequency | % | Frequency | % |
| Beclometasone | Yes | 89 | 73.6 | 68 | 56.2 | 157 | 64.9 |
|  | No | 32 | 26.4 | 53 | 43.8 | 85 | 35.2 |
| Adherence to beclometasone | Adherent | 73 | 82.2 | 13 | 19.1 | 87 | 55.1 |
|  | Non adherent | 16 | 17.8 | 55 | 80.9 | 171 | 44.9 |
| Salbutamol puff | Yes | 116 | 95.9 | 119 | 98.3 | 235 | 97.1 |
|  | No | 5 | 4.1 | 2 | 1.7 | 7 | 2.9 |
| Oral Salbutamol | Yes | 20 | 16.5 | 42 | 34.7 | 62 | 25.6 |
|  | No | 101 | 83.5 | 179 | 65.3 | 180 | 74.4 |
| Oral prednisone | yes | 17 | 14.0 | 35 | 28.9 | 52 | 21.5 |
|  | No | 104 | 86.0 | 86 | 71.1 | 190 | 78.5 |
| Theophylline | Yes | 2 | 1.7 | 9 | 7.1 | 11 | 4.5 |
|  | No | 119 | 98.3 | 112 | 92.6 | 231 | 95.5 |
| Concurrent medication | Yes | 27 | 22.3 | 63 | 52.1 | 90 | 37.2 |
|  | No | 94 | 77.7 | 58 | 47.9 | 152 | 62.8 |
| Types of concurrent medication | Anti acids | 11 | 40.7 | 25 | 39.7 | 36 | 40.0 |
|  | Antibiotics | 12 | 44.4 | 10 | 15.9 | 22 | 24.4 |
|  | Anti histamines | 4 | 14.8 | 7 | 11.1 | 11 | 12.2 |
|  | Anti  hypertensive | 3 | 11.2 | 20 | 31.7 | 23 | 25.6 |
|  | Others**^1^** | 1 | 3.7 | 3 | 4.8 | 4 | 4.4 |
| **Others^1^** Anti-thyroids, highly active retroviral therapy, Anti- pain | | | | | | | |

# Anti- asthmatic drug combinations factors

# Beclomethasone puff+salbutamol puff was the most anti-asthmatic drug combination among controls 64(52.9%) and cases 47(38.8%). Salbutamol puff + Oral salbutamol was the second prescribed anti-asthmatic drug combination [5.8% of controls and 18.2% of cases].

# Table S2: Combination ant-asthmatic drugs among study participants in Jimma University Medical Center, South West Ethiopia, 2017

|  | Control | | Case | | Total | |
| --- | --- | --- | --- | --- | --- | --- |
|  | Frequency | % | Frequency | % | Frequency | % |
| Salbutamol puff | 18 | 14.9 | 5 | 4.1 | 23 | 9.5 |
| Salbutamol puff + salbutamol po | 7 | 5.8 | 22 | 18.2 | 29 | 12.0 |
| Beclometasone puff + salbutamol puff | 64 | 52.9 | 47 | 38.8 | 111 | 45.9 |
| Beclometasone puff+ salbutamol puff + salbutamol po | 9 | 7.4 | 8 | 5.8 | 16 | 6.6 |
| Predinsolone po + salbutamol puff | 5 | 4.1 | 8 | 6.6 | 13 | 5.4 |
| Predinsolone po + salbutamol puff + salbutamol po | 3 | 2.5 | 9 | 7.4 | 12 | 5.0 |
| Predinsolone po + salbutamol puff + beclometasone puf | 8 | 6.6 | 13 | 10.7 | 22 | 8.7 |
| Other combinations | 7 | 5.8 | 10 | 10.4 |  |  |
| Other: predinsolone only, prednisone +beclometasone puff, theophylline po, only, theophylline salbutamol puff only, theophylline salbutamol puff only, salbutamol po, theophylline beclometasone puff, salbutamol puff | | | | | | |

# Socio-demographic and patient related factors associated with asthma control

Among socio-demographic and patient related factors, respondents within the age group of >55years [COR=6.12; 95% CI=2.97-12.65; p< 0.001], rural residence [COR=2.76; 95%CI=1.64-4.66; p<0.001], previous smoker [COR=6.529; 95%CI=2.96-4.37; P<0.001], current smoker [COR=6.35; 95%CI=2.03-9.80; P=0.001], poor knowledge about asthma[COR=30.57; 95%CI=13.95-17.01; P<0.001], negative attitude towards asthma[AOR=3.56; 95%CI=2.07-6.12; P<0.001] were significantly associated with poorly controlled asthma.

Table S3: Socio demographic and patient related factors associated with poorly controlled asthma in Jimma University in South West Ethiopia, 2017

| Variables | | Control N=121 | Case  N=121 | COR(95%CI) | p- value |
| --- | --- | --- | --- | --- | --- |
|  |  | n (%) | n (%) |  |  |
| Sex | Male | 64(52.9) | 54(44.6) | 1.00 |  |
|  | Female | 57(47.1) | 67(55.4) | 1.39(0.81-2.33) | 0.190 |
| Age of the respondents | 18-34 | 40(33.1) | 16(13.2) | 1.00 |  |
|  | 35-54 | 52(43.0) | 34(28.1) | 1.64(0.83-3.45) | 0.180 |
|  | >55 | 29(24.0 | 71(58.7) | 6.12(2.97-12.65) | <0.001 |
| Residence | Urban | 72(59.5) | 42(34.7) | 1.00 |  |
|  | Rural | 49(40.5) | 79(65.3) | 2.76(1.64-4.66) | <0.001 |
| Smoking status | Never | 108(89.3) | 68(56.2) | 1.00 |  |
|  | Previously | 9(7.4) | 37(30.6) | 6.529(2.96-4.37) | <0.001 |
|  | Current | 4(3.3) | 16(13.2) | 6.35(2.03-9.80) | 0.001 |
| Knowledge about asthma | Good | 112(92.6) | 35(28.9) | 1.00 |  |
|  | Poor | 9(7.4) | 86(71.1) | 30.57(13.95-17.01) | <0.001 |
| Attitude about asthma | Positive attitude | 89(73.6) | 53(43.8) | 1.00 |  |
|  | Negative attitude | 32(26.4) | 68(56.2) | 3.56(2.07-6.12) | <0.001 |

**Diseases and drug related factors associated with poorly controlled asthma.**

Among drug and diseases related variables, moderate asthma [COR=19.33; 95% CI=7.43-52.0; P<0.001], severe asthma [COR=90.22; 95%CI=28.65-87.59; P<0.001], comorbid conditions [COR=6.71; 95%CI=2.98-15.13; P<0.001], none users of ICS [COR=2.17; 95%CI=1.26-3.72; P= 0.005], non adherence to ICS [COR=22.96; 95%CI=10.07-52.71; P<0.001],were strongly associated with poorly controlled asthma on bivariate analysis.

Table S4: Diseases and drug related factors associated with poorly controlled asthma in Jimma University, South West Ethiopia, 2017

| variables | | Control N=121 | Case N=121 | COR(95%CI) | P-value |
| --- | --- | --- | --- | --- | --- |
|  |  | n (%) | n(%) |  |  |
| Severity of asthma | Mild | 70(57.9) | 5(4.1) | 1.00 |  |
|  | Moderate | 42(37.9) | 58(47.9) | 19.33 (7.43-52.05) | < 0.001 |
|  | Severe | 9(7.4) | 58(47.9) | 90.22(28.65-87.59) | < 0.001 |
| Comorbid condition | No | 113(93.4) | 82(67.8) | 1.00 |  |
|  | Yes | 8(6.6) | 39(32.2) | 6.71(2.98-15.13) | < 0.001 |
| ICS(beclometasone) | Yes | 90(74.4) | 68(56.2) | 1.00 |  |
|  | No | 31(25.6) | 55(43.8) | 2.16(1.26-3.72) | < 0.001 |
| Adherence to ICS | Adherent | 76(84.4) | 13(19.1) | 1.00 |  |
|  | None  adherent | 14(15.6) | 55(80.9) | 22.96(10.00-52.43) | < 0.001 |
